# Strategies to Enable Transformation in Medical Education: Faculty and Trainee Development in Competence By Design

**DOI:** 10.5334/pme.960

**Published:** 2024-02-06

**Authors:** Adelle R. Atkinson, Cynthia Abbott, Anna Oswald, Andree Boucher, Rodrigo B. Cavalcanti, Jason R. Frank, Linda S. Snell

**Affiliations:** 1 System of Specialties, Royal College of Physicians and Surgeons of Canada, Ottawa, ON, Canada; 2 Division of Rheumatology, Department of Medicine, Faculty of Medicine and Dentistry, University of Alberta, Edmonton, AB, Canada; 3 Competency Based Medical Education, University of Alberta, Edmonton, AB, Canada; 4 Royal College of Physicians and Surgeons of Canada, Ottawa, ON, Canada; 5 Department of Medicine (Division of endocrinology), Université de Montréal, Montréal, QC, Canada; 6 Department of Medicine, Temerty Faculty of Medicine, University of Toronto, Toronto, ON, Canada; 7 The HoPingKong Centre, University Health, Network, Toronto, ON, Canada; 8 Department of Emergency Medicine, and Director, Centre for Innovation in Medical Education Faculty of Medicine, University of Ottawa, Ottawa, ON, Canada; 9 Medicine and Health Sciences Education, Institute of Health Sciences Education and Department of Medicine,McGill University, Montreal, QC, Canada

## Abstract

Transformative changes in health professions education need to incorporate effective faculty development, but few very large-scale faculty development designs have been described. The Royal College of Physicians and Surgeons of Canada’s Competence by Design project was launched to transform the delivery of postgraduate medical education in Canada using a competency-based model. In this paper we outline the goals, principles, and rationale of the Royal College’s national strategy for faculty and resident development initiatives to support the implementation of Competence by Design. We describe the activities and resources for both faculty and trainees that facilitated the redesign of training programs for each specialty and subspecialty at the national level, as well as supporting the implementation of the redesign at the local level. This undertaking was not without its challenges: we thus reflect on those challenges, enablers, and the lessons learned, and discuss a continuous quality improvement approach that was taken to iteratively inform the implementation process moving forward.

## Introduction

Faculty development is a powerful tool that contributes to improved teaching and learning and can promote innovation and organizational change [1], especially for competency-based education [2]. Faculty development was thus a major focus of the Royal College of Physicians and Surgeons of Canada (hereafter referred to as the Royal College) strategy for the national implementation of Competence By Design, with the goals of facilitating national curricular redesign and achieving systems changes in postgraduate specialty training across all specialties at the national level.

**Figure 1:**
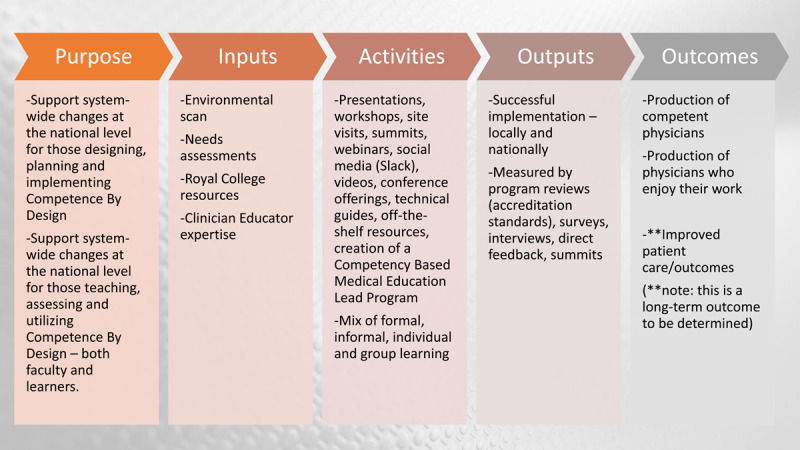
Logic Model used for Competence By Design (CBD) implementation [4].

Competence by Design (CBD) is a transformative change project of the Royal College aimed at redefining specialty training in Canada using a competency-based approach. From the beginning of the implementation of CBD, its success depended, in part, on supporting numerous widely distributed invested groups to make changes associated with the new curricular paradigm [3]. In particular, the Royal College recognized that it could help support the broad scope of national and local change by partnering with the medical schools to build capacity across multiple partner groups and organizations. Therefore, using a logic model (purpose, inputs, activities, output, outcomes) (Figure 1) incorporating an environmental scan and other needs assessments (inputs), a number of interrelated faculty development initiatives (activities) were developed and implemented [4].

For CBD, the authors define faculty development as “capacity building initiatives designed to assist target audiences to effectively fulfill their multiple roles in a competency-based residency system”. The target audiences and partners in the CBD project included the national specialty committees (responsible for standard setting and assessment at the national level for each discipline), postgraduate medical education (PGME) offices at each medical school, residency training program directors (PDs), front-line faculty teachers, and trainees. The Royal College recognized the need for capacity-building initiatives to assist in developing each individual’s roles in implementing and sustaining change. This challenge was confounded by the diversity of contexts within and between medical schools and programs offering postgraduate training nationwide.

The bundle of activities offered was informed by Steinert’s quadrant model [1] and included; presentations (synchronous and asynchronous), workshops, site visits, summits, webinars, the use of social media such as Slack, videos, conference offerings, technical guides, off-the-shelf resources, and the creation of a CBME Lead program at each medical school. It must be emphasized that trainees have traditionally not been a target audience for faculty development, but early on it was recognized that they were an essential group for whom to build capacity. As such, the Royal College iteratively adapted the logic model’s inputs and resultant activities to include trainees. This paper reviews the strategies used in the logic model, and the barriers, and enablers encountered in adopting a national approach to faculty and trainee development to achieve successful CBD implementation.

## Designing a faculty development plan for implementation of Competence by Design: goals, principles, and rationale

CBD was a multi-year, multi-partner curricular design and systems change, with the first cohorts of programs starting implementation in 2017. The broad scope of this national-level PGME initiative required interventions that leveraged a wide range of strategies to address the heterogeneity of partners’ resources and needs. Overall, the following principles underpinned the faculty development strategy for the CBD initiative.

### Clarify the overall purpose

The goal of the national faculty development strategy was to support the system-wide changes at the national level as Canadian postgraduate training programs transitioned to a competency-based curriculum. Through a bundle of faculty development initiatives, the Royal College and the medical schools, through a model of shared responsibility set out to:

- enhance the knowledge and build the skills of those responsible for designing, planning, and implementing CBD, particularly PDs and CBME Leads at all medical schools; and
- enhance the knowledge and build the skills of those who would teach, learn, and assess in a CBD system, including clinical teachers and trainees.

### Provide a variety of learning activities to account for different needs, contexts, and preferences

Given the number of medical schools (n=17), disciplines (n=67), and close to 1000 individual programs across Canada, there were vastly different needs, resources, and capacity available to support CBD. The local culture, the size of the program, and the level of access to local expertise and resources were critically important factors related to a program’s approach to and success in implementing CBD. Through an environmental scan and several needs assessments, it became clear that a variety of learning options was needed to approach this heterogeneity.

The Royal College acknowledged that the implementation of CBD would add to the workload of faculty members, program administrators, and trainees, who were already extremely busy. As such, the CBD faculty development strategy emphasized activities and resources that people could easily access, use, and adapt. These ready-to-use faculty development resources were key to the Royal College’s strategy and to those developed at many of the medical schools in Canada.

Guided by Steinert’s four quadrants [1], there was a mix of formal, informal, individual, and group learning activities. Capacity-building efforts included a variety of supports that have traditionally been associated with formal group and individual learning (e.g., workshops and courses, published literature, manuals). While these were quite useful, faculty development for CBD also emphasized other ways of building capacity such as informal, work-based, micro, and just-in-time faculty development. The choices of faculty development modalities for the different pieces of implementation work, were informed by feedback following the delivery of different sessions, and evolved significantly over time. For example, it became clear that webinars were highly valued by partners to allow them to engage in a dialogue with Royal College educators as well as colleagues nationally, to understand the resources and how to use them, and to share practical tips and tricks. This helped to build community engagement and momentum.

### Support program level leaders to contextualize and sustain the change

In Canada, PDs oversee the entire planning and implementation of the postgraduate training program for their own specialty or subspeciality at a particular medical school, including recruitment, curriculum development, development of assessment strategies, provision of trainee support, and faculty development. They are trusted collaborators and advisors in their programs. PDs are well positioned to engage their colleagues and to lead meaningful change within their program, including changes to the overarching philosophy of learning, teaching, and assessment. For this reason, supporting PDs was seen as critical to the successful implementation of CBD. Fortunately, the Royal College has well-established connections to PDs, most directly through existing Royal College specialty committees.

Specialty committees for each discipline, which includes all the PDs in the specialty, participated in a uniquely designed CBD faculty development initiative. It involved three multi-day CBD design workshops held over approximately 1.5–2 years. These workshops initially began about 24 months before a discipline’s trainees officially started CBD training. The workshops were the primary forum for designing new CBD standards and helping to prepare PDs to facilitate change in their programs by engaging their clinical colleagues, trainees, and program administrators [5].

With the first few cohorts of specialties that participated in the workshops, faculty developers experienced challenges in covering everything that was required for a smooth transition to CBD within a two-year window. This became apparent through iterative feedback sought from the specialties through their assigned clinician educator, Pulse Check surveys, Specialty Committee chairs, and discussion with institutions’ national CBME Leads. It was also evident in data from ongoing monitoring of specialty implementation regarding the ability to achieve the required benchmarks. Many PDs left the CBD Design workshops with a solid understanding of the rationale for CBD, but they required additional support outside the workshops to build their knowledge and expertise in the “how” of CBD and the effective application of the design in their home programs. For this reason, in 2019–2020, the Royal College focused on extending the timeline of support from 24 months to almost 48 months, which included extending support beyond the launch of CBD for each specialty, to support, ensure success and iteratively gather information about the implementation experience that would help other programs who were earlier on in the process. This extended support model included opportunities to reinforce and encourage the ongoing development of the PDs between the CBD design workshops, after the CBD design workshops, and after the launch of CBD through new CBD booster sessions, designed to support programs post-launch. These practical sessions focused on implementation, helping PDs map out an implementation plan and providing specific resources for each section of the faculty development map. Importantly, participants shared resources and their experiences of what worked, and what didn’t work, with their peers. The booster sessions also introduced asynchronous online learning modules, new teaching tools, and synchronous virtual learning events. Examples from this repository include a slide deck on running a Competence Committee, a module on learning the basics of coaching over time, and a two-page infographic on the 10 steps to mapping a CBD curriculum. Importantly, this extended support model also allowed the Royal College to learn what was or was not working and to iteratively design further faculty development tools. This information was also incorporated into the Program Evaluation strategy [6].

### Partner with others to encourage meaningful and sustainable change

While PDs were identified as being vital to CBD implementation, it was clear that local program change could not be the responsibility of a single individual. In 2016 each medical school in Canada identified a CBME Lead to support their local programs and guide the implementation process for CBD at the University level, specific to their context using their unique knowledge of local needs and barriers. The Royal College depended on, collaborated with, and provided support to the CBME Leads to help extend their local implementation efforts. Through a formal collaborative initiative, and formal and informal feedback, the Leads helped to inform the work of the Royal College’s faculty developers, allowing the Royal College to focus on the largest and most common challenges. Regular meetings included timely and frank conversations to address challenges and gaps, to discuss opportunities for improvement and to co-create solutions where appropriate.

Together, the Royal College and the faculties of medicine across the country set out to ensure that the learning and development needs of all groups were understood and supported. Faculty development was treated as a shared responsibility between the Royal College (at the national level) and each medical school (at the local level) with extensive support for both levels of educational activities from the Royal College. Program champions, including the PDs and CBME Leads, supported clinical teachers, trainees, and program administrators through the transition to CBD. The Royal College partnered with those educators to build trust and support and to extend local efforts through co-creation of approaches and resources.

### Ask for feedback: identify and respond to needs

The decision to roll out CBD in a cohorted fashion over several years [3, 5] was motivated in part by the understanding that the needs and experiences of early cohorts could iteratively inform the design of the support and resources offered to later cohorts. It was complex to identify the needs of key groups in the first years of CBD, as many of the CBD concepts were new and continued to evolve as the educators learned from experience. In the earliest stages of the project, the Royal College worked with the first two disciplines, Medical Oncology and Anesthesiology, to understand and address their emerging needs. These disciplines played a critical role in highlighting what areas required support, and helped in the drafting and refining of content as it was created to support the disciplines that followed. This CQI approach led to a large increase in the number of directed resources and support over time. Fortunately, PDs were keenly aware of the needs of their learners and teachers, allowing them to play a critical role in identifying local needs and gaps in support. The Royal College and CBME Leads used these data to address the gaps, often through co-creation as well as the sharing of new tools and resources. The early adopters were also key in demonstrating the heterogeneity of learning needs and contexts, which ultimately informed the Faculty Development content and strategies used for later cohorts.

As more disciplines transitioned to CBD, the Royal College implemented formal surveys and interviews to help to understand evolving needs. The Royal College also received formal and informal reports from invested groups, which helped validate or refine the approaches through iterative change.

### Produce customizable just-in-time learning and teaching

A ‘just-in-time’ approach to some of the faculty development components was key to the Royal College’s faculty development strategy and to many strategies developed locally at each medical school in Canada. Early on, the Royal College’s structure of delivering resources put the onus on the end user to seek out the content they needed. This delivery model assumed that the users were highly motivated to search for content and that they knew three key things: what they needed, when they needed it, and where to get it.

Royal College-initiated CBD implementation surveys and other direct feedback showed that these assumptions about the delivery model and the knowledge of invested groups were not universally accurate and demonstrated a gap in knowledge between the resources and supports the Royal College offered and the ones that faculty needed and could easily find. In order to address this, in 2020, the target audiences were stratified on the basis of their “stage” of CBD implementation, so that relevant content could be pushed to them when they needed it. For example, after the synchronous virtual implementation booster sessions, a list of the top 10 resources that had been discussed was sent to participants to address their current needs, including resources on competence committees, trainee orientation, growth mindset, and curriculum mapping. In addition, certain resources were curated to be ready to be pushed to the PDs in each specialty as they met certain milestones in their journey toward CBD launch (https://www.royalcollege.ca/ca/en/cbd/cbd-tools-resources.html).

### Identify priority targets for CBD faculty development

**Figure 2:**
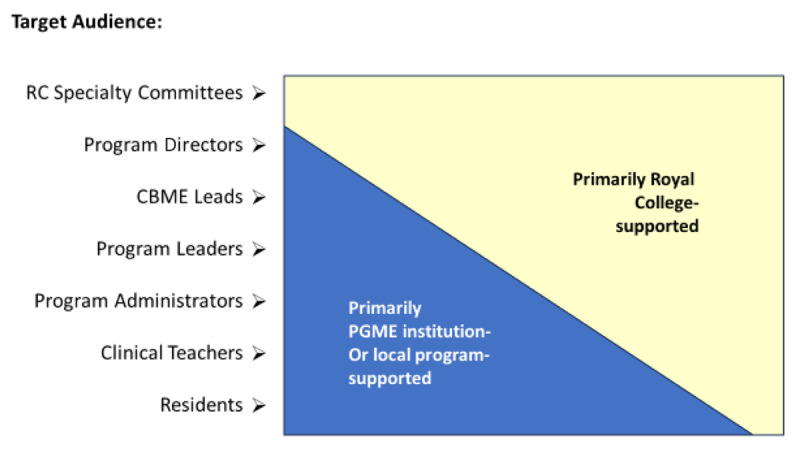
Priority targets for Royal College faculty development in the implementation of Competence by Design.

Given the scope of change associated with CBD, it was not surprising that key groups had different needs and concerns. While national specialty committees (including PDs), CBME Leads and PGME deans typically received direct support from the Royal College, other university faculty (including clinical teachers, program administrators, deans, department chairs, and division and department heads) and trainees often worked directly with their local support teams. Some challenges were institution-specific and others were more common nationally. Depending on the needs of the key groups and the opportunity for meaningful contact, this led to some of the targeted CBD faculty development being provided by the Royal College (Figure 2).

In other cases, the support was delivered locally by the medical schools, with local efforts being enhanced by a variety of Royal College resources including templates and tools (Table 1).

**Table 1:** Examples of resources created for faculty development in the implementation of Competence by Design (CBD).


RESOURCES FOR CBD CONTENT (LEARN)	RESOURCES FOR CBD FACULTY DEVELOPMENT (TEACH)

Webinars Example: CBD 101	Webinars Example: How to run your competence committee

Implementation materials Example: CBD Implementation Planner	Booster sessions aligned with workshops Example: Implementation of CBD – where should you be?

Slide decks Example: Mapping the EPAs to your curriculum	Competence Committee modules Example: How to set up a competence committee

Technical guides and policies Example: EPA Observation Forms	Coaching modules Example: Engage your faculty in WBA and coaching

Tip sheets Example: CBD Residents: Key things you need to know.	Slide decks (modifiable to be used by Program Directors responsible for CBD implementation – “off the shelf”) Example: Introduction to CBD for faculty

	*The Meantime Guide* – a guide to opportunities for programs to engage in while waiting to begin their CBD design Example: Preparing faculty to take on the role of coaching.


It became clear early on that one of the keys to efficient and effective faculty development was to utilize these established connections to strategically support the change.

### CQI Cycles for Longitudinal Faculty Development

The use of a CBD faculty development logic model allowed the Royal College to evaluate whether faculty development initiatives were achieving their goals. In addition, a continuous quality improvement approach to faculty and trainee development in CBD was used. Input from multiple sources (feedback from post activity evaluations, formal surveys, summits, interviews etc.), about many activities and resources provided data that allowed the iterative development of resources and activities. There are still challenges with making data-informed approaches, given the long delay in getting robust program evaluation data and the confounders to the key indicators.

Over time, the Royal College observed and tracked that the focus of CBD support requests began to shift from the ‘basics of CBME’ to ‘how to ensure quality implementation’ and to the desire for information on how the design of CBD would benefit learners, programs, and most importantly patients.

## What about the trainees? Change management beyond faculty

Trainees are clearly an integral part of the PGME system, but traditional faculty development activities rarely target learners. In fact, one of the most important lessons learned early in the process was the importance of preparing trainees for the changes involved in CBD. This was recognized as a clear gap in the design, and a key for success. It is clear that building skills around change management for learners, in this case related to CBD implementation, is critical and should be built into the overall approach [7]. Engaging the trainees as the “consumer” and partnering with them allowed them to contribute to the process. This was done directly with the trainees, or trainee organizations and indirectly via the PDs and CBME Leads.

Early feedback from newly launched CBD programs provided direction for this revised process. Trainees in early cohorts said that they felt underprepared for this change and thought that if they had been armed with information about the goals, design, and process of CBD, and their roles within it, they could have helped move the implementation process forward. In some ways the trainees were well positioned to champion and support their program’s faculty members through this change, especially in the early days of the launch.

This early feedback led the Royal College implementation teams to develop resources that PDs could use “off the shelf” to orient their trainees to CBD. The goal was to make these resources accessible, practical, efficient, and easy for PDs to use. Table 2 provides examples of resources for the development and support of trainees.

**Table 2:** Examples of resources created for trainee development in the implementation of Competence by Design (CBD).


TRAINEE DEVELOPMENT RESOURCES

A Resident’s Orientation to CBD

Resident Guide: What you need to know

CBD for Residents: Key things you need to know

Residents: Competence Committees

Learning the Basics of Coaching over Time

Growth Mindset Development


It was paramount that trainees understood both the goals and rationale for the change (i.e., improved patient care through ensuring competence in graduating learners) and the CBD design for their program. Armed with this information, they could more easily engage with their local curriculum and be aware of their own responsibilities (e.g., obtaining work-based assessments at regular intervals, initiating coaching conversations with their supervisors, employing a growth mindset, understanding the role of the Competence Committee). Through ongoing trainee surveys and feedback from PDs and other faculty involved in CBD, the tools evolved iteratively.

All the resources were made available through a single source on the CBD website https://www.royalcollege.ca/ca/en/cbd/cbd-tools-resources.html and were pushed directly to PDs at the appropriate time in implementation. There were multiple opportunities for PDs and other faculty members to attend webinars to be introduced to each of the tools and learn how to use them.

## Challenges, enablers, and lessons learned

Given the scope and scale of the change associated with CBD, it is unsurprising that there have been numerous faculty development challenges since initiating the approach. Each of these challenges, iteratively informed the input phase of the logic model such that activities could be modified to achieve the output and outcomes desired.

Challenges were specifically identified through a variety of sources including an environmental scan and needs assessments according to the inputs phase of the logic model. These included the CBD leads regular interaction with the Royal College, regular PD townhalls, resident summits, webinars, meetings with established partner groups, Royal College Touch Points and Pulse Checks, feedback from CBD workshops and formal resident reports from national groups [4]. The resident groups, in particular, provided very clear feedback about the challenges of CBD implementation as seen through their lens [8, 9, 10]. Reflections on this feedback informed further phases of the project, and led to changes in activities to address the concerns moving forward using this CQI approach.

The top five challenges are listed in Table 3. These include gaps in faculty members’, trainees’, and other partners’ understanding and engagement; lack of time and resources for program leaders to implement CBD; heterogeneity of needs; difficulty getting the right support to the right people; and culture change to a growth mindset. The first two challenges we described related to user groups’ understanding as well as time/resources issues, both of which have been described in the literature [11, 12]. The other challenges we encountered reflect more of the process and system issues related to a large scale conceptual and curricular change initiative [13, 14]. Further, Table 3 includes strategies that the Royal College educators found successful in ddressing these challenges and the lessons learned.

**Table 3:** Commonly encountered challenges, strategies, and lessons learned in large-scale faculty development initiatives in Competence By Design (CBD).


CHALLENGE	RESPONSE	LESSONS LEARNED AND ONGOING STRUGGLES

1. *Gaps in the engagement in and understanding of CBD by faculty, trainees and other partners*	Provide resources and guidance that are consistent and comprehensive Encourage ongoing, repeated, regular, bite-sized consumption of CBD information Incorporate CBD boosters into regular meetings and training times Tailor approach to the specific discipline, program, and relevant clinical workflow Identify trusted champions to support rollout	Orientation to CBD is a continuous process, not a one-time event Balance repetition versus new onboarding versus advanced content where there may be short leadership cycles (e.g., PD terms) Expect that groups may not initially know what they need; with experience, faculty developers become better able to anticipate needs

2. *Lack of time and resources for program leaders to implement CBD*	Partner with schools to create local champions Advocate on behalf of schools for local resources to build capacity and share tasks to free up time for key program leaders (e.g., arrange for groups of programs at a single school to learn together about designing a Competence Committee) Create or curate both comprehensive and bite-sized content options to address specific key needs (e.g., competence committees, EPA observations, coaching) Create content and resources that are easy to access, can be shared, require little effort to use, and can be adapted locally Leverage existing educational events to introduce and reinforce key CBD concepts	Be mindful of change fatigue and variations in capacity, especially when competing major changes come up (pandemic, accreditation visits, transition to electronic medical records etc.) Time challenges need regular attention; the goal is that these challenges will become less prominent as CBD processes are developed and refined and as culture change evolves

3. *Heterogeneity of needs among programs, specialties and schools*	Clear communication, trust, negotiation, and flexibility with respect to the heterogeneity are required to coordinate national- and local-level faculty development initiatives	Recognize that capacity to adopt the CBD design varied widely and each program may have had a different starting point

	Listen to the needs of partners and tailor the approach on the basis of their unique needs and expectations Maintain resource capacity to be agile and responsive to changing and varied needs	Accept that not all partners need or want your support While this initially took significant time and effort, the ability to be flexible and adapt to heterogeneity eventually became a strength of the system

4. *Difficulty getting the right support and resources to the right people at the right time*	Stratify support and resources by groups (e.g., by stage of transition or role) Curate and push content to targeted groups at anticipated times of need Build networks that welcome and onboard new members Celebrate quick wins and champions	When creating resources, plan times to pause and ensure they are relevant, accessible, and organized in a way that users can find and access them Anticipate turnover in leadership roles (PDs, CBME Leads, etc.) Be mindful of overwhelming users with resources that are not directly relevant

5. *Changing the culture of teaching and assessment toward a growth mindset*	Emphasize the teaching and feedback benefits of EPAs and CBME for trainees, beyond their role in assessment Shrink the change: provide clear direction on what is changing and what is staying the same Share best practices to demonstrate successes	Be aware of behaviours and practices that may undermine growth mindset principles (e.g., trainees only seeking a WBA when they feel as though they will be “entrusted,” which will turn low-stakes assessments into high-stakes assessments and encourage a culture in which trainees do not benefit from ongoing coaching to achieve competence) Carefully consider how other factors are shaping this culture change (data presentation in portfolios, hidden curriculum around actions of competence committees, etc.) Be patient: meaningful culture change takes time and effort and occurs at varying paces in different contexts


*Abbreviations: CBD* = competence by design; *CBME* = competency based medical education; *EPA =* entrustable professional activity; *WBA* = workplace-based assessment.

## Discussion

CBD is a systemic change in the complex ecosystem of medical education in Canada. In this paper, we have described how Steinert’s faculty development model, as a design framework, and the logic model, as an evaluation framework, iteratively guided the implementation of a national faculty development strategy. This strategy encompassed a complex, nationwide medical education system and leveraged partnerships, co-creation, and the incorporation of feedback in a closed-loop CQI approach.

This model reflects recent perspectives on faculty development approaches including formal and informal initiatives for both individuals and groups [1]. The Royal College’s faculty development initiatives reflect Steinert’s quadrants; for example, booster sessions, CBD workshops, webinars, and sessions at the International Conference on Residency Education align with Steinert’s quadrant of formal group approaches, whereas the online modules on coaching and competence committee cases fit with the quadrant of formal learning aimed at individuals. Peer coaching from a PD colleague from a cohort that transitioned to CBD earlier also aligns with an individual approach, which can be formal or informal. Communities of practice (in the group/informal quadrant) were explicitly enabled, for example, for Competence Committee chairs (the Competence Committee chairs forum) or program administrators (a program administrator conference). Mentoring was done by the CBME Leads. Although PDs learn “by the nature of their responsibilities” (the individual/informal quadrant), this learning was facilitated by resources such as technical guides and tip sheets. Although the use of social media was not mentioned by Steinert [3] it was an important part of the Royal College’s faculty development strategy to build awareness about key resources and to facilitate buy-in.

The importance of trainee development in contributing to successful implementation of CBD should not be underestimated: it led to a value-added experience for trainees. Empowering residents to be agents, even champions, of change improved not only their own engagement in the process but the quality of faculty development for others [15].

The outcomes described in this article required a deliberate framework and design as well as a commitment to nurturing the social and communication elements for sustained engagement. Encouraging key partners to use a variety of communication channels (from the level of the PD through to the level of the PGME offices) to provide input around what they needed, and what was or was not working. Ongoing attention to early results using the logic model to pivot, adjust, and fill gaps was crucial to success. Encouraging a growth mindset at all levels from the institution to the individual allowed all to learn about the change and contribute to the outcomes.

The outcomes of this national faculty development initiative may in part reflect Kotter’s eight steps for leading change [16]. The urgency of providing faculty development arose from the implementation of CBD, a radically new curricular model. Working with partners, agreeing on a goal and direction, and communicating this were essential elements. Removing obstacles was challenging, and as with any change of this magnitude, challenges were encountered. These included resistance and a lack of buy-in to a new educational model, lack of resources for implementing it, and gaps in engagement, among others. We outlined some strategies used to address these challenges. The logic model provided *short-term* outcomes such that faculty development initiatives could be modified iteratively. The current faculty development program is designed to maintain momentum and anchor the change, however the implementation of CBD continues to be a work in progress, and faculty development will continue to be a key step in achieving its goals.

## Disclaimer

The views and opinions expressed in this article are those of the authors and do not necessarily reflect the official policy or position of the Royal College of Physicians and Surgeons of Canada (“Royal College”). Information in this article about Competence by Design (“CBD”), its implementation and related policies and procedures do not necessarily reflect the current standards, policies and practices of the Royal College. Please refer to the Royal College website for current information.
